# Corticotropin-releasing hormone inhibits autophagy by suppressing PTEN to promote apoptosis in dermal papilla cells

**DOI:** 10.1080/07853890.2025.2490823

**Published:** 2025-04-12

**Authors:** Wenzi Liang, Xiuwen Chen, Na Ni, Chutong Zhuang, Zhiying Yu, Ziqing Xu, Yingshi Li, Changmin Lin, Keng Huang

**Affiliations:** a Department of Histology and Embryology, Shantou University Medical College, Shantou, PR China; b Department of Neurology, First Affiliated Hospital of Shantou University Medical College, Shantou, PR China; c Physical Examination Center, Second Affiliated Hospital of Shantou University Medical College, Shantou, PR China

**Keywords:** Hair loss, hair regrowth, chronic unpredictable mild stress, PI3K/AKT/mTOR, hypothalamic–pituitary–adrenal axis

## Abstract

**Background:**

Stress-related hair loss is on the rise, largely due to escalating levels of stress-related corticotropin-releasing hormone (CRH) through poorly defined mechanisms. CRH-mediated activation of corticotropin-releasing hormone receptors (CRHRs) on dermal papilla cells (DPCs) is a likely cause of stress-related hair loss. The aim of the study is to elucidate the key mechanisms of alopecia caused by CRH and provide potential new targets for the treatment of stress-related hair loss.

**Methods:**

4D label-free quantitative proteomics of DPCs and the chronic unpredictable mild stress mouse (CUMS) model were used to explore the relationship and mechanism between CRH, DPCs and hair regeneration.

**Results:**

CRH initially downregulated PTEN to suppress autophagy, leading to DPC apoptosis. Overexpression of PTEN enhanced autophagy and mitigated CRH-dependent DPC apoptosis. CRH inhibited PTEN and activated the PI3K/AKT/mTOR pathway, whereas rapamycin inhibited this pathway and activated autophagy, consequently lowering apoptosis, suggesting that increased susceptibility to apoptosis is caused by decreased autophagy. CUMS-induced hair growth disruption is accompanied by an increase in CRHRs and a decrease in PTEN levels within the dermal papilla. Intracutaneous injection of CRH impeded hair regeneration and decreased PTEN in mice, concurrent with inhibition of autophagy and increased apoptosis.

**Conclusions:**

These findings indicate that PTEN loss coupled with PI3K/AKT/mTOR-mediated autophagy inhibition and apoptosis in DPCs is a key mechanism of stress-related hair loss induced by CRH and suggests that topical activation of PTEN or enhancement of autophagy, e.g. through rapamycin, may have a therapeutic effect on stress-induced hair loss disorders such as alopecia.

## 1. Introduction

Hair loss is a skin disorder caused by multiple factors, with psychological stress being one of the major causes [1]. A growing number of people are suffering from hair loss due to psychosocial stress, but there is currently no effective treatment [2]. The classical reaction to stress involves activation of the hypothalamic-pituitary-adrenal (HPA) axis. Stressors can all elicit a response from the HPA axis, beginning with hypothalamic release of corticotropin-releasing hormone (CRH) [3]. Evidence indicates that skin and hair follicles possess systems analogous to the HPA axis, enabling hair follicles to react to the HPA activation [4], and several studies have shown a link between the HPA axis and hair cycle [5–8]. CRH is the primary activator of the HPA axis and a key player in stress response [9]. Researches have suggested that CRH affects the hair growth cycle and may play a role in hair loss due to stress [4,7]. However, the exact mechanism by which CRH affects hair regrowth is not well understood.

Hair follicle is a miniature organ and an important preclinical research model [10,11]. Two types of CRH receptors (CRHRs), CRHR1 and CRHR2, are localized predominantly in the hair follicle dermal papilla (DP) [12], the mesenchymal region that plays a key regulatory role in hair growth. The DP is organized as a tightly knit group of specialized dermal papilla cells (DPCs) located at the hair follicle’s base, a privileged location that enables DPCs to activate and regulate the hair cycle through reciprocal interactions with hair follicle stem cells in the neighbouring epithelium [13]. Expression of CRHRs allows the DP to be responsive to CRH [4,7], indicating a potential involvement of CRH in mediating hair loss through poorly defined mechanisms.

CRH interferes with cellular levels of autophagy [14] and increases apoptosis [15], both of which are strongly associated with psychological stress-related disorders [16]. Autophagy and apoptosis, being interrelated processes, are vital for maintaining physiological homeostasis of hair growth, as well as pathogenesis of hair loss [11]. Therefore, stress could be linked to hair loss through CRH-mediated regulation of DPC survival and function through autophagy and apoptosis. Exploring the specific pathophysiological mechanisms by which psychological stress affects the hair growth cycle can contribute to the development of new targeted therapeutic strategies. Thus, we analyzed and identified the differentially expressed proteins on CRH-treated DPC, using 4D label-free quantitative proteomics, and found that phosphatase and tensin homolog deleted on chromosome 10 (PTEN) loss may be a key mechanism of hair loss. PTEN can regulate cellular energy expenditure along with its capability to modulate proliferation and survival [17]. PTEN inactivates PI3K/AKT/mTOR-dependent metabolic pathways to enhance autophagy [18,19], as well as intervenes in cellular apoptosis [20].

Altogether, we hypothesized that CRH induces stress-related hair loss by inhibiting DPC autophagy and increasing DPC apoptosis through modulating PTEN and PI3K signalling. The aim of this study was to identify the mechanism of stress-related hair loss induced by CRH and elucidate the regulatory mechanisms of related signalling cascades. Through a clearer understanding of the role of stress hormones in the development of hair loss, this work may provide a theoretical basis for the treatment of stress-related hair loss. These findings have the potential to establish a scientific foundation for the development of treatments aimed at stress-induced hair loss, thereby pioneering new pathways to improve the quality of life for those suffering from hair loss. Hair follicle is an important preclinical research organ [10,11]. Studying the growth cycle and regeneration mechanism of hair follicle provides a theoretical basis for studying the biological issues of cell proliferation, tissue interaction and organ regeneration. This basic knowledge is essential for understanding the pathologic processes and regenerative repair of other organs.

## 2. Materials and methods

### 2.1. Cell culture

Human scalp tissues were obtained from the Second Affiliated Hospital of Shantou University Medical College, with the approval of the Second Affiliated Hospital of Shantou University Medical College Ethics Committee (approval number: 2024–38). All participants provided their written informed consent for the use of these tissues in research. This work has adhered to the Declaration of Helsinki. DPCs underwent initial culture and subsequent subculturing following established protocols [21]. DMEM (Gibco, Grand Island, NY, USA) containing 10% fetal bovine serum (Invitrogen-Gibco) was used to continuously culture DPCs in an incubator at 37 °C, 5% CO_2_ and 90% humidity.

DPCs were treated with 10^−7^ M CRH (Absin, abs45151738, designated as the CRH group) to simulate the stressed state, and total protein was extracted at different times for biochemical detection [22,23]. Fresh culture-medium containing CRH was replaced every day to ensure CRH sustained effect. To explore the specific receptors engaged by CRH, DPCs were pretreated with the CRHR1 antagonist antalarmin (Anta, MCE, HY-124475, designated as the CRH+Anta group) or CRHR2 antagonist antisauvagine-30 (aSvg-30, MCE, HY-P1107A, designated as the CRH+aSvg-30 group) for 1 h, and then exposed to CRH. To inhibit both CRHRs, a non-selective CRHR1/CRHR2 antagonist, astressin (Ast, GLPBIO, GC16218, designated as the CRH+Ast group), was administered 1 h prior to CRH treatment. To inhibit the PI3K/AKT/mTOR signalling pathway, DPCs in the CRH group were treated with rapamycin (Rap, MCE, HY-10219, designated as the CRH+Rap group). When dimethylsulphoxide was used as the solvent, the dilution ratio was between 0.001%∼0.0001%.

### 2.2. 4D Label-free quantitative proteomics

Three individual patient-originated (Scalp trauma; ages 37, 46 and 57) DPCs were used for the studies. DPCs, derived from the frontal scalp of 3 women, were treated with normal medium (Ctrl group) and medium containing 10^−7^ M CRH (CRH group) for 72 h. Proteins were extracted and digested with trypsin for 4D label-free quantitative proteomics (Shanghai Applied Protein Technology Co., Ltd.) [24].

### 2.3. Engineering of PTEN- overexpressing DPCs

DPCs overexpressing PTEN were constructed by lentiviral transduction (EF1α/GFP&Puro). CRH-treated DPCs and untreated DPCs were infected with noncoding control lentivirus (Lv-NC) or PTEN-encoding lentivirus (Lv-PTEN) to establish three groups: Lv-NC, CRH+Lv-NC, and CRH+Lv-PTEN groups. On the 7th day after transduction, the transduction efficiency (% GFP-positive) was assessed by fluorescence microscopy, and the expression of PTEN was assessed by western blotting. In the CRH+Lv-NC and CRH+Lv-PTEN groups, CRH was added to the DPCs on the fourth day following transduction.

### 2.4. Cell counting kit-8 (CCK-8) assay

DPCs were seeded at a density of 2.5 × 10^3^ cells/well in 96-well plates and allowed to attach overnight. Then medium was changed to fresh medium with or without CRH. After incubation for 0, 1, 2, 3, 5 and 7 days, 10 μL of CCK-8 assay reagent was added to each well for 1 h, then a spectrophotometer was used to record the optical density (OD) at a wavelength of 450 nm.

### 2.5. Western blot analysis

Total DPC protein was extracted and separated on SDS-PAGE gels, then transferred to PVDF membranes (Millipore, USA). After blocking, the entire uncropped membrane was incubated for 30 min with mouse anti-GAPDH (internal control) antibody. After three washes, the membrane was incubated overnight at 4 °C with primary antibody. The next day, after three washes, membrane was then incubated with secondary antibody for 1 h at room temperature. Visualization of ­protein bands was carried out using enhanced chemiluminescence detection reagent in conjunction with X-ray film (Tanon, China). Primary antibodies included: PTEN (CST, 9559 T), LC3 (CST, 12741 T), P62 (Abcam, ab109012), Beclin1 (Abcam, ab207612), caspase3 (CST, 9662S), caspase9 (CST, 9502 T), BAX (Proteintech, 60267-1-Ig), Bcl2 (Proteintech, 12789-1-AP), β-catenin (Abcam, ab32572), LEF1 (Abcam, ab53293), phospho-PI3K (P-PI3K, Abcam, ab191606), PI3K (Abcam, ab302958), phospho-AKT (P-AKT, Abcam, ab81283), AKT (Abcam, ab179463), phospho-mTOR (P-mTOR, Abcam, ab109268), mTOR (CST, 2983S), phospho-ULK1 (P-ULK1, CST, 14202S), ULK1 (CST, 8054 T), P53 (CST, 2527 T), P21 (CST, 2947 T), GAPDH (Proteintech, 60004-1-Ig).

### 2.6. Annexin V-PI apoptosis assay

Apoptotic DPCs were examined through an Annexin V-PI double-staining method. DPCs were treated with 10^−7^ M CRH for 72 h. Then, single-cell suspensions of DPCs were obtained using trypsin without EDTA, followed by staining in accordance with the Annexin V-Alexa Fluor 647/PI Kit protocol (4 A Biotech, FXP023). The stained cells were subjected to flow cytometric analysis 15 min post-staining. Early apoptosis represents the initial phase of programmed cell death, characterized by the externalization of phosphatidylserine on the cell membrane. Late apoptosis is the subsequent phase, marked by the loss of membrane integrity. Annexin V-positive cells are indicative of early apoptosis, and PI-positive cells represent late apoptotic or necrotic cells [25].

### 2.7. TEM

DPCs were collected and washed with precooled PBS, fixed with 2.5% glutaraldehyde at 4 °C for more than 4 h, followed by a 1 h post-fixation in 1% osmium tetroxide. After being embedded and selected, DPCs underwent double staining with uranyl acetate and lead citrate and were then observed using a JEM-F200 electron microscope.

### 2.8. Animals

The Shantou University Medical College Animal Ethics Committee granted approval for all conducted animal studies (approval number: SUMCXM2024-416). All animals were treated in accordance with the principles of the ARRIVE guidelines. Five male C57BL/6 mice, 7 weeks old, were randomly used per group (Charles River, Beijing, China).

A chronic unpredictable mild stress mouse (CUMS) model was used to explore the relationship between stress and hair growth, and to explore the role of CRH in the pathogenesis of stress-related hair loss. Before modelling, the dorsal hair of each mouse was shaved, and chemically removed during the telogen (resting phase, during which the bulge remains dormant). Hair removal induces synchronized anagen (expansion phase, during which the bulge of the hair follicle produces a new bulb) of hair follicles over the depilated area [26]. After hair removal, control mice were kept as usual, whereas mice designated for CUMS were exposed to a variety of stressors for 21 days. CUMS mice were exposed to two different alternating stressors every day, including soiled bedding (12h), noise (12h), restraint (2h), hanging by the tail (6 min), strobe lighting (12h), deprivation of water or food (12h), tail pinching (1 min), swimming in 4 °C/40 °C water (5 min) and vibration (10 min) [27]. Following 21 days of stress, control mice and CUMS mice were subjected to a battery of anxiety-related behavioural tests to evaluate their mental state and anxiety-like behaviours. These tests included the open field test, where mice were placed in an open field arena (50 cm × 50 cm) for 5 min; the forced swim test, which consisted of a 2-min acclimation period followed by a 4-min formal testing period; and the sucrose preference test, which assessed the mice’s preference for a 1% (w/v) sucrose solution [27].

To further explore the influence of CRH on hair growth, in mice that had completed hair removal, saline was injected intracutaneously into the dorsal skin of control (Ctrl) mice, and CRH was injected intracutaneously into untreated mice (CRH mice, 10 ng/mouse once a day for 14 consecutive days). To block CRHRs, mice were administered astressin intracutaneously 1 h prior to receiving CRH injections (100 μg per kg, CRH+Ast mice). Hair regeneration in each group was observed over 14 days.

### 2.9. Haematoxylin and eosin (HE) staining and immunofluorescence staining

Localization and quantification of CRHR1, CRHR2 and β-catenin in cultured DPCs were performed by immuno-fluorescence microscopy. DPCs were fixed in 4% paraformaldehyde for 20 min and blocked and permeabilized with quick blocking buffer (Beyotime, P0260) containing TritonX-100 for 10 min. DPCs were incubated with primary antibodies at 4 °C overnight. The next day, DPCs were incubated with fluorescently labelled secondary antibodies for 1 h at room temperature. Images were obtained with a confocal microscope at 400× magnification.

Dorsal skin samples of mice were obtained for histological examination. Tissues were preserved in 4% paraformaldehyde solution for 8 h and encased in paraffin. Subsequently, skin sections were prepared at a thickness of 4 micrometres for staining with HE. Stained sections were examined under bright field microscopy. Localization and quantification of CRHR1, CRHR2, PTEN, LC3 and cleaved caspase-3 in mouse skin were performed by multiplex immunohistochemistry. Samples were treated with fluorescently labelled antibodies, and images of stained slides were obtained with a confocal microscope at 100× and 400× magnifications.

### 2.10. Statistical analysis

All data are presented as the means ± SD from at least three independent experiments. Data analysis was conducted utilizing GraphPad Prism version 8.0 (GraphPad Software, USA). Differences between groups were evaluated using an unpaired t-test. Statistical significance was determined when the probability value was less than 0.05 (*p* < 0.05).

## 3. Results

### 3.1. CRH initially suppresses autophagy and subsequently induces apoptosis in DPCs

**Figure 1.**
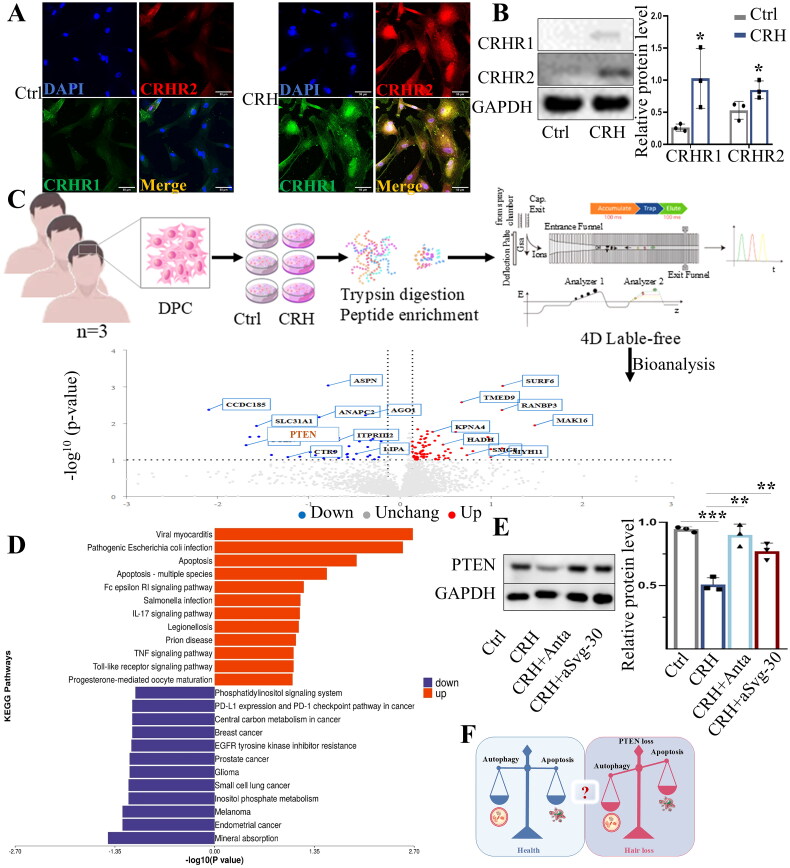
CRH downregulates the expression of PTEN in DPCs. A. Representative fluorescence images of CRHR1 and CRHR2 of control (ctrl) DPCs and DPCs exposed to 10^−7^M CRH for 12 h (CRH). Scale bars 50 μm. B. Western blot analysis of CRHR1, CRHR2 in DPCs, CRH treatment upregulated the expression of CRHR1 and CRHR2 (*n* = 3). C. Strategy for 4D label-free quantification of proteomics for DPCs treated with 10^−7^ M CRH for 72 h. Volcano plot of the differentially-expressed proteins between CRH-treated and control human scalp DPCs. Red dots indicate increased proteins, and blue dots indicate decreased proteins in CRH-treated DPCs (*n* = 3 independent biological samples per group). D. Enriched KEGG pathways. E. Western blot analysis of PTEN in cultured DPCs (12 h, *n* = 3). F. Hypothesis on the mechanism of stress-related hair loss. Data are means ± SD. *p < 0.05, **p < 0.01, ***p < 0.001, ****p < 0.0001. N.S., not significant.

To confirm whether DPCs can respond to CRH in this system, immunofluorescence techniques were used to determine the expression intensity and location of CRHR1 and CRHR2 on CRH-treated or untreated DPCs. CRHR1 fluorescence intensity was increased after exposure to 10^−7^ M CRH for 12 h. Similarly, CRHR2 fluorescence intensity was increased in DPCs following CRH treatment (Figure 1A). Western blotting confirmed that CRH treatment of DPCs increased levels of CRHR1 and CRHR2 (Figure 1B). Recent studies have highlighted that autophagy can be impaired by CRHR activation [7,14]. Western blot analysis of microtubule-associated protein light chain 3-II (LC3II), a protein that drives the formation of autophagosomes, indicated that autophagy in DPCs was not significantly altered 2 h after CRH exposure. However, a decrease in autophagy was observed following 6, 12, and 18 h of CRH treatment, with the most pronounced reduction occurring at the 12-h mark (Supplemental Figure S1). Changes in cell viability after CRH treatment was measured by CCK8 assay. The results showed no significant change in DPC viability on days 1 and 2, but a significant decline in DPC viability was observed on day 3 (72 h) (Supplemental Figure S2). To further explore the mechanism of action of CRH on DPCs, DPCs from three different patients were treated with CRH for 4D label-free quantitative proteomics analysis, and key differentially expressed proteins were identified (Figure 1C). KEGG pathway analysis indicated that many DPC signalling pathways were altered after CRH treatment, with the apoptosis signalling pathway being enriched within the dataset (Figure 1D). The volcano plot in Figure 1C shows a significant reduction of PTEN, a key protein that activates autophagy and regulates apoptosis [19]. Western blotting confirmed that CRH treatment of DPCs for 12 h reduced PTEN levels, which could be prevented by either blocking CRHR1 with antalarmin or blocking CRHR2 with antisauvagine-30 (Figure 1E). Thus, we hypothesized that PTEN loss, inhibition of autophagy and the induction of apoptosis are linked in the regulation of DPCs and hair cycle by CRH (Figure 1F).

**Figure 2.**
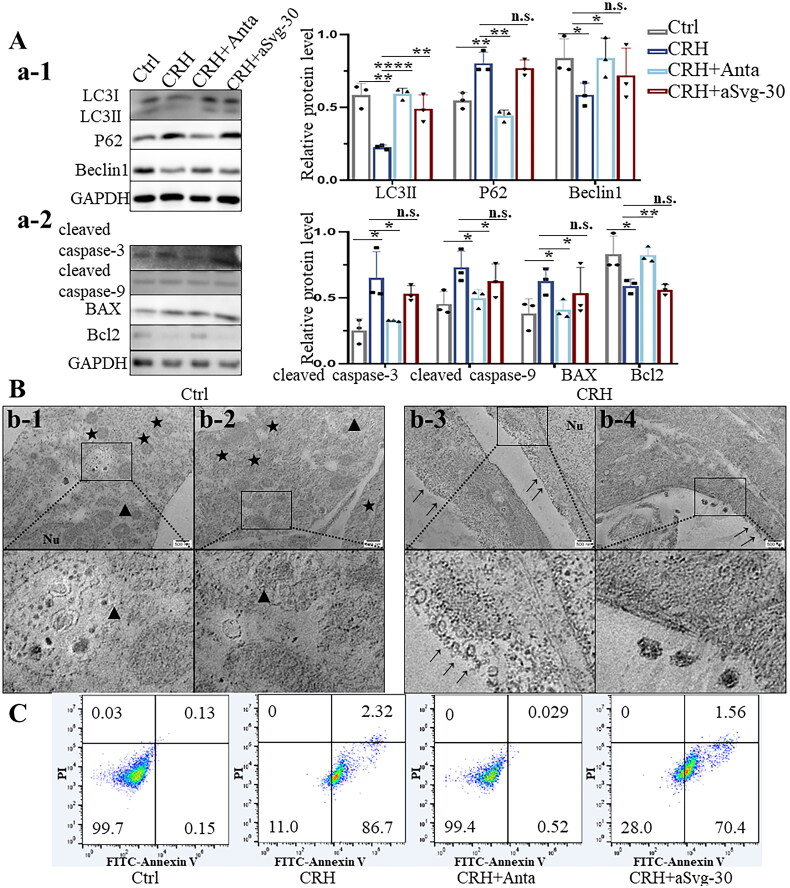
CRH inhibits autophagy, and consequently triggers apoptosis in DPCs. A. a-1. Effect of CRH on autophagy-related proteins of DPCs (12 h, *n* = 3). Decreased expression of LC3-II and increase of P62 level in CRH-treated DPCs indicated inhibition of autophagy. a-2. Effect of CRH on apoptosis-related DPC proteins (72 h, *n* = 3). In CRH-treated DPCs, expression of cleaved caspase-3 and cleaved caspase-9 indicates the induction of apoptosis, while increased bax and decreased Bcl-2 suggest apoptotic activity. B. TEM image of control DPCs or CRH DPCs for 72 h. Each group presents an image near the nucleus (b1, b3) and an image of the cytoplasm (b2, b4). Scale bars 500 nm. Nu, nucleus;→, damaged cytomembrane; ★, autophagosome;▲, autolysosome. C. Flow cytometric analysis of apoptotic DPCs in different groups using Annexin V-PI double staining. Data are means ± SD. *p < 0.05, **p < 0.01, ***p < 0.001, ****p < 0.0001. N.S., not significant. LC3-II, microtubule-associated protein light chain 3-II; P62, sequestosome 1; bcl-2, B-cell lymphoma 2; bax, bcl-2-associated X protein.

Western blotting was used to assess the levels of autophagy-associated proteins LC3, P62, and Beclin1 in DPCs following a 12-h treatment with CRH. Additionally, the effects of blocking CRHR1 or CRHR2 on protein expression were individually examined. CRH decreased the levels of LC3II and Beclin1 while elevating P62. In contrast, pretreatment with antalarmin not only counteracted the decrease in LC3II and Beclin1, but also diminished P62 levels, whereas pretreatment with antisauvagine-30 solely mitigated the decrease in LC3II (Figure 2a-1). This implies that a 12-h CRH treatment decreases autophagy in DPC through activation of CRHR1, but not CRHR2.

Corresponding to the results of the CCK8 assay (Supplemental Figure S2), an increase in apoptotic proteins, including cleaved caspase-3, cleaved caspase-9, and BAX, coupled with a decrease in anti-apoptotic Bcl2, was observed at 72 h after CRH treatment. Pre-treatment with antalarmin, but not antisauvagine-30, prevented CRH-mediated apoptosis (Figure 2a-2).

**Figure 3.**
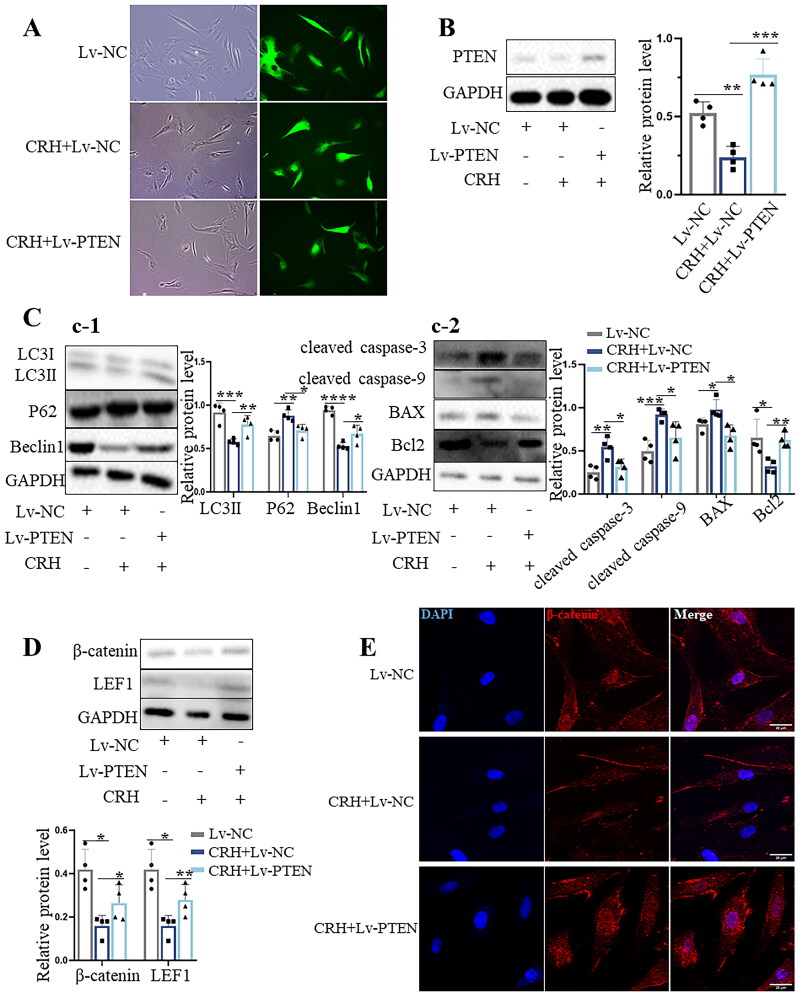
Overexpression of PTEN in CRH-treated DPCs enhances autophagy and reduces apoptosis. A. Representative images of GFP expression in control DPCs or CRH-treated DPCs. Scale bars 50 μm. B. Western blot analysis of PTEN in DPCs in different groups (*n* = 4). C. C-1. Western blot analysis of autophagy-related proteins of DPCs in different groups (*n* = 4). C-2. Western blot analysis of apoptosis-related proteins of the DPCs in different groups (*n* = 4). D. Western blot analysis of β-catenin and LEF1 of DPCs in different groups (*n* = 4). E. Representative fluorescence images of β-catenin of DPCs in different groups. Scale bars 25 μm. Data are means ± SD. *p < 0.05, **p < 0.01, ***p < 0.001, ****p < 0.0001.

**Figure 4.**
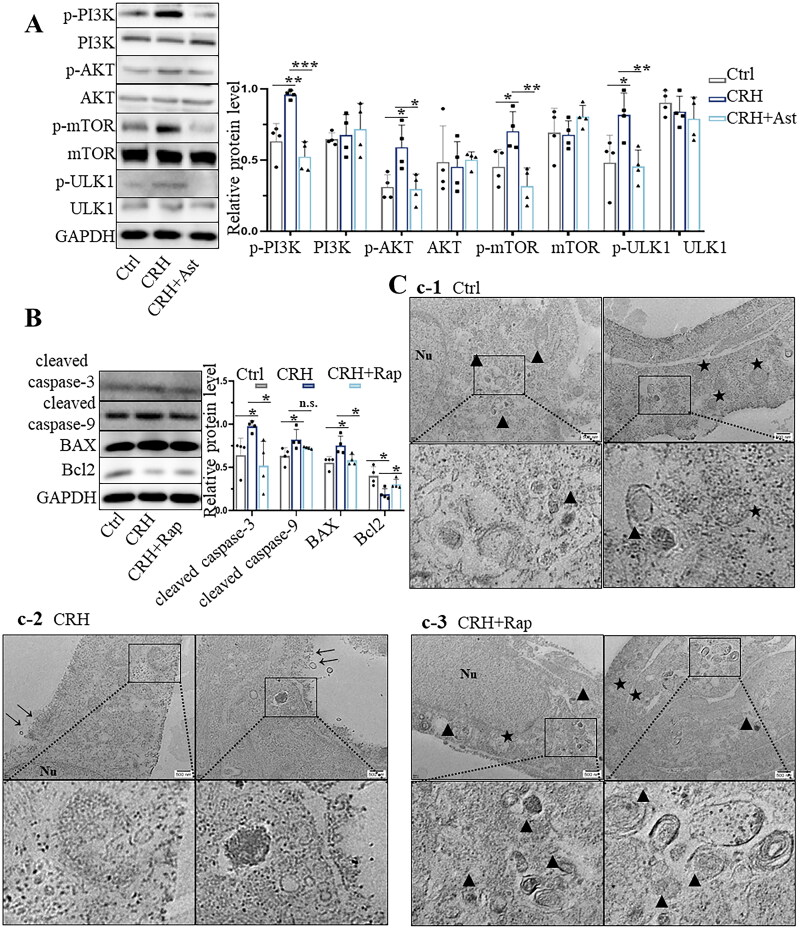
Inhibition of CRH-mediated PI3K/AKT/mTOR signaling pathway activation stimulates autophagy and decreases apoptosis. A. Western blot examination of autophagy-related proteins in control (ctrl), CRH-treated and CRH+ast-treated DPCs (*n* = 4). B. Western blot examination of apoptosis-related proteins in control, CRH-treated and CRH+rap-treated DPCs (*n* = 4). C. TEM images of control (C1), CRH-treated (C2) and CRH+rap-treated (C3) DPCs. Scale bars 500 nm. Nu, nucleus;→, damaged cytomembrane;★, autophagosome;▲, autolysosome. Data are means ± SD. *p < 0.05, **p < 0.01, ***p < 0.001, ****p < 0.0001.

TEM was used to characterize the morphological characteristics of DPCs at the ultrastructural level. TEM findings showed that in the control group, DPCs exhibited an intact cytomembrane and nuclear envelope, and an abundance of autophagosomes and autophagolysosomes within the cytoplasm (Figure 2b-1, Figure 2b-2). In CRH-treated DPCs, typical apoptotic morphological characteristics were observed with TEM, including cytomembrane and nuclear envelope blebbing and apoptotic bodies, as well as a reduction in the number of autophagosomes in the cytoplasm (Figure 2b-3, Figure 2b-4). Antagonizing CRHR1 with antalarmin increased the number of autophagosomes and autophagolysosomes within CRH-treated DPCs. The plasma membrane appeared clear and intact, with no signs of apoptotic bodies. Upon CRHR2 antagonism with antisauvagine-30, the plasma membrane exhibited blebbing and fragmentation, and there were only a few autophagosomes and autophagolysosomes within CRH-treated DPCs (Supplemental Figure S3). Annexin V-PI staining confirmed that 72-h CRH treatment induced apoptosis in DPCs, predominantly resulting in early apoptosis. However, CRH treatment had minimal impact on late apoptosis and necrosis of DPCs. As expected, antalarmin rescued the early apoptosis caused by CRH, whereas antisauvagine-30 did not reverse the apoptosis (Figure 2C).

Collectively, 4D label-free quantitative proteomics revealed that CRH reduces PTEN expression in DPCs and triggers apoptosis. Further studies confirmed that CRH initially suppresses autophagy and subsequently induces apoptosis in DPCs in a manner mainly mediated through CRHR1, with the suppression of autophagy preceding the initiation of apoptosis, and this process is likely facilitated by the downregulation of PTEN.

### 3.2. Overexpression of PTEN in CRH-treated DPCs can stimulate autophagy and mitigate apoptosis

Decreased autophagy caused by the absence of PTEN could lead to increased susceptibility of DPCs to apoptosis. Lv-NC and Lv-PTEN lentiviruses were used to infect DPCs. On the 7th day post transduction, GFP expression was detected by fluorescence microscopy, confirming successful lentiviral transduction (Figure 3A). Western blotting further showed that PTEN levels were decreased in CRH-treated Lv-NC DPCs compared to control Lv-NC DPCs, whereas PTEN levels remained high in CRH-treated Lv-PTEN DPCs compared to CRH-treated Lv-NC DPCs (Figure 3B). We next characterized the autophagy and apoptosis levels of these cells. Western blotting revealed that overexpression of PTEN led to an upregulation of LC3II and Beclin1, as well as a reduction in P62 levels within CRH-treated Lv-PTEN DPCs (Figure 3C-1). This suggests that PTEN overexpression prevents the reduction in autophagy mediated by CRH treatment. In addition, overexpression of PTEN also reduced the levels of cleaved caspase-3, cleaved caspase-9 and BAX, and increased the expression of Bcl2 (Figure 3C-2). These results indicate that PTEN mitigates CRH-triggered apoptosis of DPCs.

The Wnt/β-catenin signalling pathway is well established to be essential for the periodic growth and regeneration of hair follicles. Activation of Wnt/β-catenin signalling is closely related to the hair follicle-inducing activity of DPCs [28]. Western blotting showed that CRH inhibited Wnt/β-catenin signalling, based on decreased β-catenin and Lef1 expression, whereas restoration of PTEN levels in DPCs retained Wnt/β-catenin pathway component expression (Figure 3D). Immunofluorescence staining showed that β-catenin was expressed in the cytoplasm and nucleus of Lv-NC DPCs, and there was aggregation in the nucleus. The fluorescence intensity of β-catenin in the nucleus and cytoplasm was weak in CRH-treated Lv-NC DPCs. Stronger fluorescence intensity was observed in CRH-treated Lv-PTEN DPCs, and β-catenin had translocated into the nucleus, suggesting good ability to induce hair follicle regeneration.

Collectively, these experiments demonstrate that CRH suppresses autophagy in DPCs, thereby inducing apoptosis, a response attributable to the deficiency of PTEN. PTEN overexpression restores autophagy in DPCs and attenuates apoptosis.

### 3.3. CRH activates the PI3K/AKT/mTOR signalling pathway, while rapamycin inhibits this pathway and activates autophagy, consequently lowering apoptosis

PTEN inactivates PI3K/AKT/mTOR signalling [18], and CRH is suspected to disrupt the balance between autophagy and apoptosis by activating the PI3K/AKT/mTOR signalling pathway in DPCs.

Detection of DPCs at 12 h following addition of 10^−7^ M CRH showed activation of the PI3K/AKT/mTOR signalling pathway, based on elevation of p-PI3K (P85α), p-AKT (S473), p-mTOR (Ser2448) and p-ULK1 (Ser757) (Figure 4A). Pretreatment with astressin, to block activation of CRHRs, inhibited CRH-mediated activation of the PI3K/AKT/mTOR signalling pathway in CRH+Ast-treated DPCs. We used TEM and western blot to examine autophagy following addition of rapamycin to inhibit the PI3K/AKT/mTOR signalling pathway. Western blotting showed that rapamycin counteracted the CRH-mediated decrease in LC3II (Supplemental Figure S4). The TEM results showed that the autophagy level of DPCs significantly increased after inhibition of mTOR compared with the control group and CRH-treated group (Figure 4C1,C2,C3). After inhibition of PI3K/AKT/mTOR pathway and activation of autophagy by rapamycin, western blotting and TEM results showed a reduction in DPC apoptosis by rapamycin. Rapamycin treatment reduced the levels of cleaved caspase-3, cleaved caspase-9 and BAX, and increased the expression of Bcl2. Compared with DPCs treated with CRH alone, TEM revealed a significant proliferation of autophagosomes in DPCs treated with CRH and rapamycin, along with a notable absence of apoptotic features (Figure 4B, C). Annexin V-PI staining also confirmed that rapamycin rescued the early apoptosis caused by CRH (Supplemental Figure S5).

Collectively, all experiments in Figure 3 suggest that CRH can regulate the autophagy of DPCs *via* PTEN and PI3K/AKT/mTOR signalling pathways, and subsequently trigger the apoptosis of DPCs, and that the increased susceptibility to apoptosis is caused by decreased autophagy, representing a key mechanism through which CRH induces abnormal hair cycling and potentially contributes to stress-induced hair loss.

### 3.4. CUMS-induced hair growth disruption is accompanied by the activation of CRHRs and a decrease in PTEN

**Figure 5.**
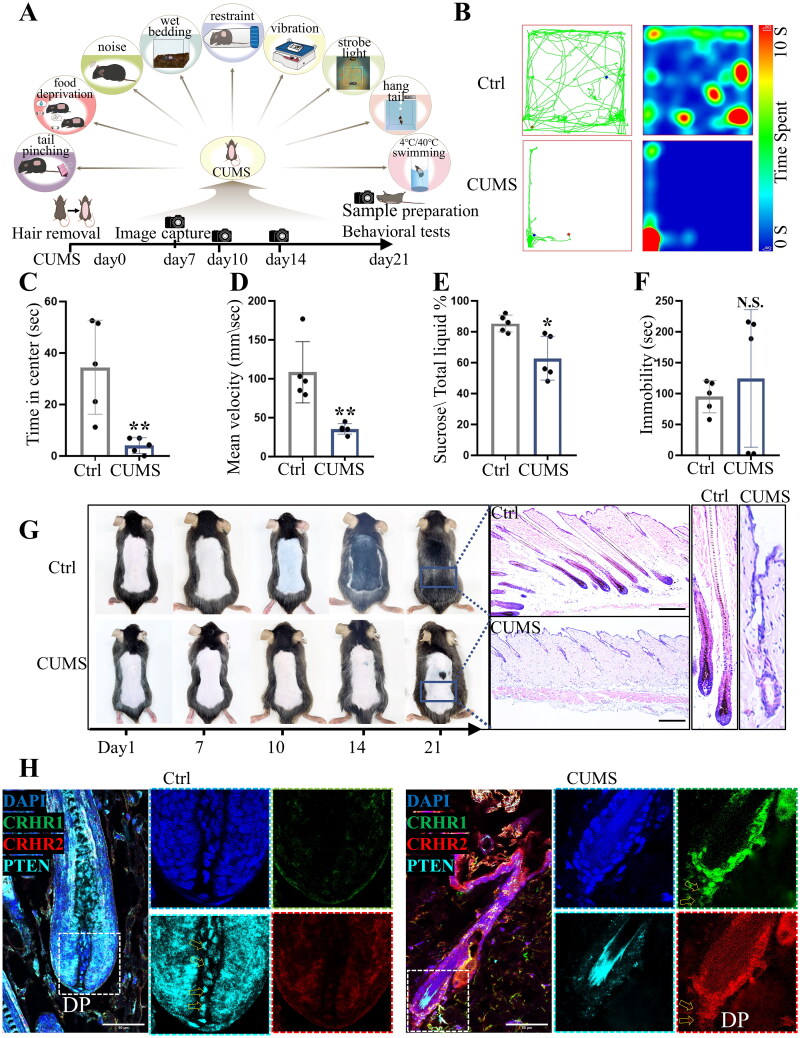
Hair regrowth delay is coincident with the activation of CRHRs and a reduction in PTEN in the DP of CUMS mice. A. Experimental design of the CUMS model, hair removal, assessment of behavior, and evaluation of hair regeneration. B. Representative trace line maps and trajectory-heatmaps of control (ctrl) and CUMS mice in the open field test. C. Duration spent in the Central zone of the open field test area. D. Average speed in the open field test. E. Sucrose preference ratio in sucrose preference assessments. F. The total duration of immobility observed in the forced swim test. G. Hair follicle regeneration of control and CUMS mice on days 1, 7, 10, 14 and 21 post shaving, and HE images at 21 days post shaving (*n* = 5 mice per group). Scale bars 100 μm. H. Representative fluorescence images of CRHR1, CRHR2 and PTEN of DP and DPCs from control and CUMS mice (day 21). Scale bars 50 μm. Data are means ± SD. *p < 0.05, **p < 0.01, N.S., not significant.

To delve deeper into the correlation between stress, CRH and hair cycling in CUMS mice, the changes of PTEN in CUMS mice were also investigated. Construction of the 21-day CUMS paradigm is shown in Figure 5A. On day 21, behavioural tests related to anxiety and depression were performed. Representative trace line maps and trajectory-heatmaps show the general activity of control and CUMS mice in the open field test. CUMS mice, in contrast to control mice, tended to stay closer to the walls, exhibited increased peripheral movement, and displayed slower movement velocities, along with a reduced overall level of locomotion (Figure 5B,D), as well as spent less time in the central area of the open field enclosure (Figure 5C). These open-field test results indicated anxiety-like behaviours in CUMS mice, characterized by diminished motor activity and a decrease in exploratory activity. Stress-induced anhedonia was assessed through the sucrose preference test. The ratio of sucrose consumption to total fluid intake was found to be lower in CUMS mice when compared to the control group, suggesting the existence of anhedonia and indicative of a depressive state (Figure 5E). In the forced swim test, three CUMS mice showed an increase in immobile time and a state of despair. Another two CUMS mice were overreactive, indicating an anxious state distinguished from despair, from which they had been struggling (Figure 5F). Behavioural test outcomes confirmed the successful establishment of the CUMS model, with CUMS mice exhibiting signs of anxiety and depression. We then used this model to explore the relationship between stress and hair regeneration, and to uncover the pathological mechanisms involved.

In contrast to the control mice, the hair cycle of CUMS mice was disrupted and anagen delayed. By the 14th day post hair removal, hair follicles of the CUMS mice were observed to be persistently in telogen, with only a minor portion of hair follicles transitioning into anagen by the 21st day (Figure 5G). Due to the involvement of multiple factors in the CUMS model, the stress responses in CUMS mice are inconsistent [29]. Some CUMS mice exhibit a severe delay in their hair cycle, while others show a mild delay (Supplemental Figure S6). In control mice, hair transitioned into anagen phase by the 10th day post hair removal, with full hair regrowth observed by the 21st day. HE staining revealed that hair follicles of control mice were robust and deeply embedded within the adipose tissue dermis, indicative of anagen, whereas the skin of CUMS mice exhibited reduced thickness, with hair follicles failing to penetrate the deep dermis, and an accumulation of immature hair follicles in telogen (Figure 5G).

Characterization, by immunofluorescence staining, of CRHR1, CRHR2 and PTEN in the mouse DP showed the fluorescence intensity of CRHR1 and CRHR2 in CUMS mice was high, indicating an increase in CRHRs. The fluorescence intensity of CRHRs was weak in the DP of control mice, while the fluorescence intensity of PTEN was high. In the DP of CUMS mice, the fluorescence intensity of PTEN decreased along with the increase in CRHRs (Figure 5H). In whole skin and hair follicles, an increase in CRHRs and a corresponding decrease in PTEN levels was detected in CUMS mice, aligning with the general alterations observed in the DP (Supplemental Figure S7). This further supports the involvement of CRH in stress-induced hair cycle disruption. Reduced PTEN levels and increases in CRHRs were observed in CUMS mice with delayed hair regeneration, which is consistent with our previous *in vitro* studies (Figures 2, 3, 4).

### 3.5. Intracutaneous injection of CRH impedes hair regeneration and decreases PTEN in DPCs, concurrent with inhibition of autophagy and increased apoptosis

**Figure 6.**
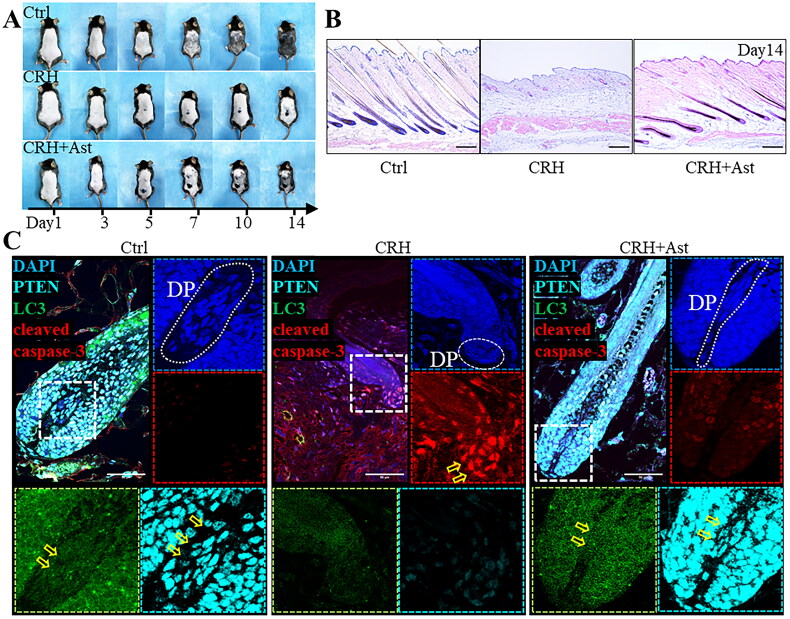
Intracutaneous CRH administration inhibits hair growth, decreases PTEN expression in hair follicles, inhibits autophagy and increases apoptosis. A. Hair follicle regeneration of control (ctrl), CRH and CRH+ast mice on days 1, 3, 5, 7, 10 and 14 post-depilation (*n* = 5 mice per group). B. HE images at 14 days post-depilation of skin from control, CRH and CRH+ast mice. Scale bars 100 μm. C. Representative fluorescence images of PTEN, LC3 and cleaved caspase-3 in the DP and DPCs from control mice, CRH mice and CRH+ast mice (D14). Scale bars 50 μm.

Subsequently, CRH was administered intracutaneously on a daily basis for a period of 14 days to mice (CRH mice). CRH administration led to a suppression of hair follicle anagen and a subsequent delay in hair regrowth compared to the control mice. In contrast, prior treatment with astressin (CRH+Ast mice) effectively prevented the CRH-induced suppression of hair follicle regeneration (Figure 6A). Histological analysis revealed that CRH suppressed hair shaft growth up through day 14. CRH mice exhibited thinner skin and possessed a reduced number of hair follicles, which were also smaller in size. In contrast, the CRH-induced obstruction of hair follicle regeneration was counteracted by pretreatment with astressin (Figure 6B, Supplemental Figure S8).

Expressions of PTEN, LC3 and cleaved caspase-3 in the DPs of control, CRH and CRH+AST mice were visualized by immunofluorescence. In the control mouse DP, PTEN and LC3 immunofluorescence was intense, whereas cleaved caspase-3 exhibited weaker fluorescence. Following intracutaneous administration of CRH, the DP exhibited reduced fluorescence intensity for PTEN and LC3, in contrast to the enhanced fluorescence intensity observed for cleaved caspase-3. Pretreatment with astressin prevented both the CRH-mediated reduction in autophagy and increased apoptosis (Figure 6C). The trend of fluorescence intensity changes for PTEN, LC3 and cleaved caspase-3 observed in whole skin was consistent with that of the DP (Supplemental Figure S9). Results of the *in vivo* experiments were consistent with the results showing that CRH can attenuate autophagy by reducing PTEN, in human scalp DPCs cultured *in vitro*, eventually leading to apoptosis. This further suggests that CRH is an important cause of stress-related hair loss, and that PTEN-mediated autophagy reduction and apoptosis activation are key mechanisms in the inhibition of hair regeneration caused by CRH.

## 4. Discussion

The objective of this research was to explore the mechanisms of stress-related hair loss induced by CRH in the DP. Through quantitative proteomics and confirmation in a mouse chronic stress model, we found that CRH induces hair loss by inhibiting autophagy through suppressing PTEN and activation of the PI3K/AKT/mTOR pathway in DPCs. A key innovation of this study, distinguishing it from prior studies, is its emphasis on elucidating the regulatory role of PTEN loss in the ability of DPCs to induce hair follicle regeneration. This work suggests that topical activation of PTEN or enhancement of autophagy, for example through rapamycin, may have a therapeutic effect on stress-induced hair loss disorders. Additionally, this method has the potential to be more targeted and less invasive than systemic therapies, thereby potentially reducing the risk of side effects. However, the major challenge of rapamycin is its immunosuppressive effect, which can lead to increased susceptibility to infections and other complications [30]. Additionally, the efficacy of rapamycin for hair regrowth and the appropriate dosing regimen both require further investigation.

Previous studies have demonstrated that acute emotional stress can cause severe localized inflammation and trigger alopecia areata, most likely by activating overexpressed CRHR2-β surrounding hair follicles. Somewhat inconsistent with previous findings, our study found that CRH requires at least 72 h for DPC apoptosis, where CRHR1 plays the predominant role compared to CRHR2. Our 14-day CRH intracutaneous injection model and 21-day CUMS model suggest that CRH is a key factor in chronic stress-induced hair loss. Compared to the negative effects of acute stress, the results of this study have more practical significance in explaining the mechanisms by which various chronic unpredictable stresses encountered in daily life lead to hair loss. In this work, antagonizing either CRHR1 or CRHR2 can restore the CRH-mediated PTEN loss. But CRH treatment decreases autophagy in DPC through activation of CRHR1, not CRHR2. Our study shows that PTEN is a key upstream regulator of autophagy, while autophagy in the downstream does not recover after antagonizing CRHR2. We speculate that there are other unknown upstream crosstalk of autophagy. The MAPK pathway, which can be activated by CRH [31], is also a key regulator of autophagy [32]. We hypothesize that the MAPK pathway may serve as an important crosstalk mechanism, which needs further exploration.

Previous studies demonstrate that during autophagy, lysosomes degrade damaged cellular components to produce precursor molecules for energy synthesis and meeting metabolic requirements. Autophagy is a protective mechanism against dangerous situations in cells [33]. In addition, prior investigators have shown that activating autophagy by rapamycin inhibits apoptosis, while suppressing autophagy by chloroquine greatly enhances T-2 toxin-induced apoptosis [34]. We find that inhibiting autophagy enhances susceptibility to apoptosis and further suggest that activation of autophagy may alleviate hair loss by reducing apoptosis.

Recent studies have shown that CRH can lead to cell aging [35]. Similarly, changes in P53 pathway-related proteins were also observed in the 4D label-free quantitative proteomics presented here, where CRH elevated expression of the aging-associated markers P53 and P21 in DPCs. Astressin pretreatment partially blocks the effects of CRH (Supplemental Figure S10A). Moreover, CRH treatment induces high levels of senescence-associated β-galactosidase (βGal) activity in DPCs in a manner that can be reduced by astressin pretreatment or rapamycin treatment (Supplemental Figure S10B). Multiple studies have shown that ­rapamycin is a reversible growth inhibitor that can slow mTOR-driven aging [36]. We also observed in this study that rapamycin reversed CRH-mediated aging (Supplemental Figure S10B). We postulate that CRH can cause the aging of DPCs and delay hair cycling, but whether this is also the mechanism of hair loss caused by CRH needs further study.

This study is subject to certain limitations, CRH was observed to reduce PTEN in DPCs, and PTEN loss is key to regulating the balance of autophagy and apoptosis. However, the specific mechanism of how CRH reduces PTEN remains unknown. Many studies have found that PTEN is lost mainly through the following ways: mutation, post-transcriptional regulation by ncRNAs, epigenetic and transcriptional regulation, phosphorylation, ubiquitination, oxidation and acetylation [37]. This study focuses on the pathophysiological mechanism of stress-induced hair loss. In addition, the study of hair follicles is a good preclinical research model to explore the role of stress hormones, autophagy, and the PTEN/PI3K/AKT/mTOR pathway in human tissue physiology, and to evaluate the efficacy and tissue toxicity of both autophagy and PI3K/AKT/mTOR pathway regulators in living human tissue. The study of the mechanism of PTEN loss is important for elucidating the pathogenesis of stress-related hair loss and will be further studied in the future. After inhibiting the PI3K/AKT/mTOR signalling pathway with rapamycin, we observed that it significant, but not completely, reversed the apoptosis induced by CRH. Therefore, we believe that the PI3K/AKT/mTOR signalling pathway plays a key role in CRH-mediated apoptosis, but there may indeed be crosstalk with other unknown signalling pathways. Further research is needed to fully elucidate these complex interactions.

Research on stress-related hair loss helps to understand how stress affects systems in the body, such as the nervous system, immune system, and skin system. These mechanisms are not only applicable to hair loss but also have potential for repair of other stress-related disorders, such as those involving wounding regeneration, neurodegenerative diseases and cardiovascular disorders, which occur under chronic psychological stress. And the research has the potential to help identify individuals who may be at high risk of developing psychiatric disorders at an early stage and might require additional psychological support. The research involves multiple disciplines such as biology, psychology and dermatology, and this interdisciplinary research approach can promote a more comprehensive understanding of diseases, including mental disorders, and advance the development of new diagnostic and treatment methods.

**Figure 7.**
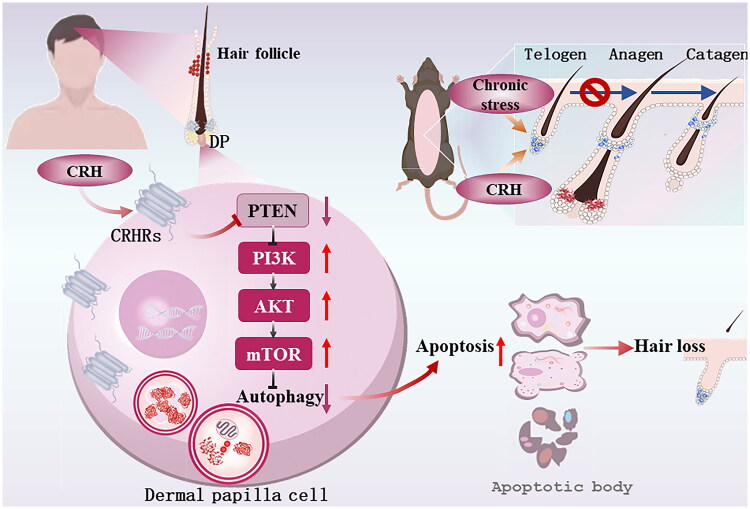
Schematic diagram of the pathophysiological mechanism of stress-related hair loss.

In summary, the present study reveals that PTEN loss in DPCs inhibits autophagy, leading to subsequent increases in apoptosis *via* activation of the PI3K/AKT/mTOR signaling pathway, and serves as an important mechanism of CRH-associated hair loss. The increased susceptibility to apoptosis is caused by decreased autophagy. Overexpression of PTEN or inhibiting the PI3K/AKT/mTOR pathway can activate autophagy to reduce apoptosis. A schematic diagram of the pathophysiological mechanism of stress-related hair loss for this study is shown in Figure 7. This work contributes to the present understanding of chronic stress-related hair loss, and further suggests that topical medication leading to activation of PTEN or improving autophagy may contribute to the treatment of stress-related hair loss.

## Supplementary Material

ARRIVE Guidelines Checklist.pdf

Supplementary material.docx

## Data Availability

Further information and requests for resources should be directed to and will be fulfilled by the lead contact, Keng Huang(huangkeng789@foxmail.com). The MS files have been deposited to the Proteome X change consortium: PXD057745.
