# Supplementary material for: Corticotropin-releasing hormone inhibits autophagy by suppressing PTEN to promote apoptosis in dermal papilla cells
Source: Ann Med. 2025 Apr 12;57(1):2490823. doi: 10.1080/07853890.2025.2490823 (PMC11995766; doi:10.1080/07853890.2025.2490823)
Supplement: Supplementary material.docx [file IANN_A_2490823_SM7486.docx]

**Corticotropin-releasing hormone inhibits autophagy by suppressing PTEN to promote apoptosis in dermal papilla cells**

Wenzi Liang^1^, Xiuwen Chen^2^, Na Ni^3^, Chutong Zhuang^4,^ Zhiying Yu^3^, Ziqing Xu^3^, Yingshi Li^3^, Changmin Lin^1*^, Keng Huang^4*^

^1^ Department of Histology and Embryology, Shantou University Medical College, Shantou, PR China.

^2^ Department of Neurology, First Affiliated Hospital of Shantou University Medical College, Shantou, PR China.

^3^ Shantou University Medical College, Shantou, PR China.

^4^ Physical examination center, Second Affiliated Hospital of Shantou University Medical College, Shantou, PR China.

^*^Correspondence author:

Changmin Lin, Department of Histology and Embryology, Shantou University Medical College, Shantou, 515041, Guangdong Province, PR China;

Tel:13829480132

Email: cocolin@stu.edu.cn

Keng Huang, Physical examination center, Second Affiliated Hospital of Shantou University Medical College, Shantou, 515041, Guangdong Province, PR China.

Tel:13502998930

Email: [huangkeng789@foxmail.com](mailto:huangkeng789@foxmail.com)

**Figure S1.** Western blot analysis of the expression levels of LC3 in cultured DPCs at 0, 2, 6, 12, and 18 hours post CRH treatment. (n=3) Data are means ± SD. *p < 0.05, **p < 0.01. N.S., not significant.

**Figure S2.** CCK8 analysis was conducted to evaluate the cell viability of cultured DPCs at 0, 1, 2, 3, 5 and 7 days post CRH treatment. (n=3) Data are means ± SD. **p < 0.01, ***p < 0.001, ****p < 0.0001.

**Figure S3.** Transmission electron microscope image of DPCs in the CRH+Anta treated group or CRH+aSvg-30 treated group. Scale bars 500 nm. Nu, nucleus;→, damaged cytomembrane;★, autophagosome;▲, autolysosome.

**Figure S4.** Western blot analysis of the expression levels of LC3 in cultured control, CRH-treated and CRH+Rap-treated DPCs. (n=4) Data are means ± SD. *p < 0.05.

**Figure S5.** Flow cytometric analysis of apoptotic DPCs in different groups using Annexin V-PI double staining.

**Figure S6.** Phenotypes and hair regeneration of 5 control (Ctrl) mice, and 5 CUMS mice on day 21 after hair removal in the CUMS model. (n=5)

**Figure S7.** Representative fluorescence images of CRHR1, CRHR2 and PTEN of hair follicles from control (Ctrl) and CUMS mice (D21). Scale bars 200 μm and 50 μm.

**Figure S8.** Phenotypes and hair regeneration of 5 control (Ctrl) mice, 5 CRH mice, and 5 CRH+Ast mice 14 days after hair removal in a CRH subcutaneous injection model. (n=5)

**Figure S9.** Representative fluorescence images of PTEN, LC3B and cleaved caspase-3 of hair follicles from control, CRH and CRH+Ast mice (D14). Scale bars 200 μm and 50 μm.

**Figure S10.** CRH increases the expression of aging-related indicators in DPCs. A. Western blot analysis of P53 and P21 in control (Ctrl), CRH-treated and CRH+Ast-treated DPCs (n=4). B. β-galactosidase staining images of DPCs in different groups. Scale bars 50 μm. Data are means ± SD. *p < 0.05.
